# CADFU for Dermatologists: A Novel Chronic Wounds & Ulcers Diagnosis System with DHuNeT (Dual-Phase Hyperactive UNet) and YOLOv8 Algorithm

**DOI:** 10.3390/healthcare11212840

**Published:** 2023-10-27

**Authors:** Syed Muhammad Ahmed Hassan Shah, Atif Rizwan, Ghada Atteia, Maali Alabdulhafith

**Affiliations:** 1 Department of Computer Science, COMSATS University Islamabad, Attock Campus, Attock 43600, Pakistan; syedmahmedhassan321@gmail.com; 2 Department of Computer Engineering, Jeju National University, Jejusi 63243, Republic of Korea; atifrizwan@jejunu.ac.kr; 3 Department of Information Technology, College of Computer and Information Sciences, Princess Nourah bint Abdulrahman University, P.O. Box 84428, Riyadh 11671, Saudi Arabia

**Keywords:** artificial intelligence, chronic wounds, foot ulcers, medical diagnosis, deep learning, DHuNeT, YOLOv8

## Abstract

In recent times, there has been considerable focus on harnessing artificial intelligence (AI) for medical image analysis and healthcare purposes. In this study, we introduce CADFU (Computer-Aided Diagnosis System for Foot Ulcers), a pioneering diabetic foot ulcer diagnosis system. The primary objective of CADFU is to detect and segment ulcers and similar chronic wounds in medical images. To achieve this, we employ two distinct algorithms. Firstly, DHuNeT, an innovative Dual-Phase Hyperactive UNet, is utilized for the segmentation task. Second, we used YOLOv8 for the task of detecting wounds. The DHuNeT autoencoder, employed for the wound segmentation task, is the paper’s primary and most significant contribution. DHuNeT is the combination of sequentially stacking two UNet autoencoders. The hyperactive information transmission from the first UNet to the second UNet is the key idea of DHuNeT. The first UNet feeds the second UNet the features it has learned, and the two UNets combine their learned features to create new, more accurate, and effective features. We achieve good performance measures, especially in terms of the Dice co-efficient and precision, with segmentation scores of 85% and 92.6%, respectively. We obtain a mean average precision (mAP) of 86% in the detection task. Future hospitals could quickly monitor patients’ health using the proposed CADFU system, which would be beneficial for both patients and doctors.

## 1. Introduction

In recent times, artificial intelligence has had a huge impact on healthcare. The use of AI-based systems for medical diagnosis is increasing day by day. In smart hospitals in particular, the use of IoT and AI is increasing tremendously. The use of machine learning, deep learning, computer vision, and the internet of things deeply impacts medical diagnosis, and it is used in every department of hospitals for computer-aided diagnosis of brain tumors, computerized diagnosis of blood cancer and colon cancer, in histopathology, breast cancer detection in mammograms, etc. [1,2]. Artificial intelligence-based intelligence systems are very helpful for medical professionals because these types of systems assist doctors in many different scenarios. For example, MRI has a lot of slices, and checking every slice is very difficult, so AI systems are very helpful for analyzing each slice very precisely in a few seconds and generating a report for the whole MRI. Similarly, in hospitals, if every day hundreds of thousands of patients come, then it is difficult for doctors to analyze every patient, and the chance of errors and mistakes is very high. The use of an intelligent system is necessary in hospitals to reduce the workload and efficiently manage patient loads.

These days, artificial intelligence is applied in every medical field, including dermatology, for skin cancer detection, wound detection, and assessments [3]. Dermatology, a specialized branch of medicine, focuses on the diagnosis and treatment of various skin-related conditions, diseases, and disorders. This field encompasses a wide array of skin issues, ranging from infections and allergies to tumors and other abnormalities. In this research paper, we study the utilization of artificial intelligence in the field of dermatology, specifically for the computer-aided diagnosis of diabetic foot ulcers (wounds).

Diabetic foot ulcers are chronic and may cause severe conditions. The foot ulcers are caused by poor blood flow and foot trauma. Any kind of internal infection or disease also causes them. These ulcers may cause traumatic infections and other life-threatening conditions if they are not treated in time. In dermatology, dermatologists are now using image analysis and pattern recognition techniques (classical and deep learning techniques) to detect these kinds of chronic ulcers. AI is very helpful in this case because using AI-based techniques is a very powerful way to detect the ulcers as well as to figure out the depth of the ulcer wounds [4,5]. Through AI algorithms, this study aims to enhance the diagnosis and management of wounds, ultimately resulting in improved patient outcomes and a reduced healthcare burden.

Diabetic foot ulcers (DFUs) pose a significant challenge in the realm of diabetes management. If inadequately addressed, this condition can result in the dire consequence of amputation. This complication exhibits a diverse presentation, with DFUs manifesting at various locations on the foot, showcasing variations in size, color, and contrast—a reflection of the underlying pathologies. However, the existing clinical strategies for DFU treatment predominantly rely on the diligence of patients and clinicians. This approach, although essential, carries notable limitations, encompassing the substantial financial burden incurred by the intricate diagnosis, treatment, and extended care associated with DFUs.

In our title, we use the phrase “CADFU for Dermatologists”, which describes a system that is beneficial to dermatologists for several reasons. First of all, it offers assistance in diagnosis and efficient screening to quickly analyze and categorize various types of wounds. Secondly, the system proves useful when dermatologists need to handle a large number of cases in less time. It can also serve an educational purpose by visually explaining computer-aided wound diagnosis. The system’s outcomes can contribute to research in the dermatology field. Additionally, automation reduces the risk of errors, even in more complex scenarios.

This research aims to develop an efficient computer-aided diagnosis system capable of accurately segmenting and detecting foot ulcers. To achieve this objective, the system utilizes the YOLOv8 algorithm, also known as “You Only Look Once”, for the detection process [6]. We use a large amount of data to train the YOLOv8 model for the wound detection task. We chose YOLOv8 because it is an advanced and more efficient version compared to all other versions of the YOLO family. YOLOv8 is more precise, fast, and capable of detecting at a multi-scale level. The main proposed system is CADFU, in which we perform two tasks: segmentation and detection. The detection task is done using the YOLOv8 model. The primary task of YOLOv8 is to accurately identify the position of the wound in an image. The detection part alone is very useful for medical professionals to deeply and efficiently analyze the patient with the help of this AI-based model.

Secondly, the research introduces the novel DHuNeT autoencoder, which stands for Dual-Phase Hyperactive UNet, and is used for segmentation. In this research, DHuNeT is specifically used for the wound segmentation task. The main task of DHuNeT is to take an image as input and map the image X to its output mask function, e.g., Y. This is done using encoder–decoder models. DHuNeT is made up of two sequentially connected UNet autoencoders. The first UNet takes the image as input; the encoder and decoder of the first UNet process the image and produce the mask. The mask is then input to the second UNet combined with the actual image. Now, learning is very fast because we use a hyperactive knowledge transfer concept. Hyperactive knowledge transfer is a process of transferring all features learned by the first UNet to the second UNet so that the second UNet can more precisely learn the output mask Y. The first UNet’s encoder transfers its features to the second UNet’s encoder. Similarly, the first UNet’s decoder transfers learned features to the second UNet’s decoder for efficiently predicting the mask. Section 3 briefly describes the proposed DHuNeT model and its functioning. The key contributions of this paper are:

1. This paper introduces CADFU, a novel computer-aided diagnosis system designed to detect and segment diabetic foot ulcers. The system demonstrates the ability to accurately identify and isolate wounds within images. The CADFU framework comprises two distinct models: DHuNeT, which focuses on segmentation, and YOLOv8, which handles detection.
2. The primary contribution of this study lies in the DHuNeT algorithm, employed for precise wound region segmentation from imaging datasets. DHuNeT comprises two sequentially stacked UNet autoencoders with hyperactive knowledge transfer, ensuring seamless information exchange from the first UNet to the second. Furthermore, the DHuNeT model finds utility beyond wound segmentation, extending its application to other medical abnormality segmentation tasks. In this paper, the second UNet is sometimes referred to as the auxiliary UNet.
3. The dataset employed in this research originates from the renowned MICCAI 2021 Foot Ulcer Segmentation(FUSeg) Challenge, a benchmark for foot ulcer segmentation [7]. Notably, our system has exhibited superior performance, outperforming others in terms of various performance metrics. This underscores the potential of our system for real-time wound diagnosis applications within smart healthcare environments. Moreover, the FUSeg Challenge represents a significant milestone in advancing wound segmentation techniques, contributing to the evolution of medical imaging technologies and diagnostic methodologies.

This paper is organized as follows. Section 2 summarizes the related literature of the topic at hand. Section 3 describes the methodologies used in the current research. Section 4 presents the obtained results and the related analysis, and Section 6 concludes the findings of the paper.

## 2. Related Work

Foot ulcers, commonly associated with diabetes mellitus, pose significant challenges due to their potential for severe complications and elevated mortality rates, including the risk of lower leg amputations. Precise extraction of morphological characteristics from these wounds is imperative for effective treatment. While visual assessment by medical experts is the conventional diagnostic method, its subjectivity and susceptibility to errors warrant alternative approaches. Enter computer-aided methods, which offer a promising solution. Leveraging deep learning, specifically convolutional neural networks (CNNs), has proven highly successful in various medical image analysis tasks, including segmentation.

In [8], authors introduce an ensemble strategy centered on two encoder–decoder CNN models, LinkNet and UNet, designed for foot ulcer segmentation. Addressing the challenge of limited training samples, they employ pre-trained weights (EfficientNetB1 for LinkNet and EfficientNetB2 for UNet), followed by further pre-training using the Medetec dataset. A spectrum of morphological and color-based augmentation techniques complements this. To elevate segmentation efficacy, their approach integrates five-fold cross-validation, test time augmentation, and result fusion. When tested on publicly available chronic wound datasets and the MICCAI 2021 Foot Ulcer Segmentation (FUSeg) Challenge, their method demonstrates state-of-the-art performance, achieving a Dice score of 88.80%. Impressively, their approach also takes the lead on the FUSeg Challenge leaderboard.

Both acute and chronic wounds, stemming from diverse causes, present substantial challenges and place a significant economic burden on global healthcare systems. Projections suggest that the advanced wound care market will surpass USD 22 billion by 2024 [9]. Wound care professionals heavily rely on image documentation for accurate diagnosis and treatment. Unfortunately, a lack of expertise can lead to erroneous wound etiology diagnoses, resulting in inadequate management and documentation. Accurately segmenting wound areas within images is a vital aspect of the diagnosis and treatment process, enabling precise measurement and quantitative assessment. Deep learning models, including those for semantic segmentation, have emerged as successful tools in image analysis.

In 2020, a study by Chuanbo et al. presented an innovative convolutional framework that leverages MobileNetV2 and connected component labeling to accurately segment wound regions within natural images [10]. An essential advantage of this model is its lightweight and computationally efficient design, maintaining a high level of performance comparable to more complex neural networks. To fortify their approach, they curate a meticulously annotated wound image dataset comprising 1109 foot ulcer images obtained from 889 patients, serving as the basis for training and evaluating the deep learning models. The effectiveness and versatility of their method are demonstrated through a thorough array of experiments and analyses encompassing a variety of segmentation neural networks.

In this context, a remarkable advancement surfaces by introducing a dataset encompassing 705 foot images. This repository not only presents a novel avenue for research but also bridges a critical gap by providing a meticulously annotated ground truth that precisely demarcates the ulcer region and its encompassing skin. This carefully outlined boundary emerges as an invaluable indicator, guiding clinicians in their assessment of ulcer progression. Complementing this contribution, a groundbreaking method surfaces, wherein a two-tier transfer learning methodology is proposed by Manu et al. [11]. Harnessing the potential of expansive datasets, this technique molds the capabilities of fully convolutional networks (FCNs) to adeptly and autonomously segregate the ulcer region and the surrounding skin. With rigorous validation through five-fold cross-validation, the ramifications of this approach become evident. The two-tier transfer learning FCN models exhibit notable success, achieving Dice similarity coefficients of 0.794 (±0.104) for the ulcer region. These outcomes herald the remarkable promise encapsulated within FCNs for DFU segmentation—a realm where their potential is poised for further amplification through the accrual of a more expansive dataset.

Burn injuries, marked by their grave implications and substantial morbidity and mortality rates, demand meticulous diagnosis and precise assessment of burn area and depth for effective treatment outcomes. The accuracy of these evaluations can often make the difference between life and death for patients. However, while employed for assessment, the existing methodologies, like the straight-ruler technique, aseptic film trimming, and digital camera photography, lack the essential attributes of repeatability and comparability.

In response to these obstacles and to refine the diagnostic procedure, strategic integration of deep learning technology arises as a revolutionary remedy. By embedding deep learning within the domain of burn diagnosis, Chong et al. introduce a semi-automated strategy that minimizes human inaccuracies, consequently enhancing the accuracy of burn evaluation [12]. This shift in approach carries the potential to completely transform burn diagnosis, establishing a uniform and dependable structure that empowers medical professionals to make well-informed treatment choices. The eventual result is the advancement of patient outcomes.

Lately, the utilization of smartphone wound image analysis has gained momentum as a viable method for evaluating healing progress and offering actionable insights to patients and caregivers between hospital visits. After segmentation, which is a crucial step in image analysis, attributes of the wound segment, such as tissue composition and wound area, can be scrutinized. The associative hierarchical random field (AHRF) represents the image segmentation issue as a graph optimization challenge.

Extracted handcrafted features are subjected to classification using machine learning classifiers. More recently, the emergence of deep learning methods has exhibited exceptional performance across a broad spectrum of image analysis tasks. Convolutional neural networks (CNNs) like FCN, UNet, and DeepLabV3 have demonstrated prowess in semantic segmentation. Despite individual experiments showcasing promising outcomes, prior research has yet to meticulously and systematically compare these approaches on a consistent dataset of substantial wound images. Additionally, the comparison between deep learning and non-deep learning wound image segmentation strategies still needs to be explored.

In [13], authors conducted a comprehensive assessment of AHRF and CNN methods (FCN, UNet, DeepLabV3), employing various metrics, including segmentation accuracy (Dice score), inference time, training data requirements, and performance across diverse wound sizes and tissue types. The study also delves into potential enhancements through image pre- and post-processing techniques. Recognizing the common limitation of limited medical images/data, the study explores the impact of dataset size on method sensitivity. The findings reveal that AHRF yields higher accuracy than UNet for small datasets (<300 images), although it falls short of the precision demonstrated by FCN and DeepLabV3. AHRF also exceeds its performance in terms of inference time.

## 3. Proposed Method

Diabetic ulcers, prevalent among chronic wounds, demand significant attention due to the potential for severe complications if not treated in a timely manner. To address this concern, our study introduces a comprehensive computer-aided diagnosis system specifically designed for foot ulcers. This system undertakes two primary tasks simultaneously: wound detection and wound segmentation. The accomplishment of these tasks relies on distinct algorithms. Advanced YOLOv8 is harnessed for precise detection, while a novel DHuNeT model is devised for accurate segmentation. The forthcoming sections provide concise insights into these algorithms’ functioning and architectural specifics.

**Figure 1:**
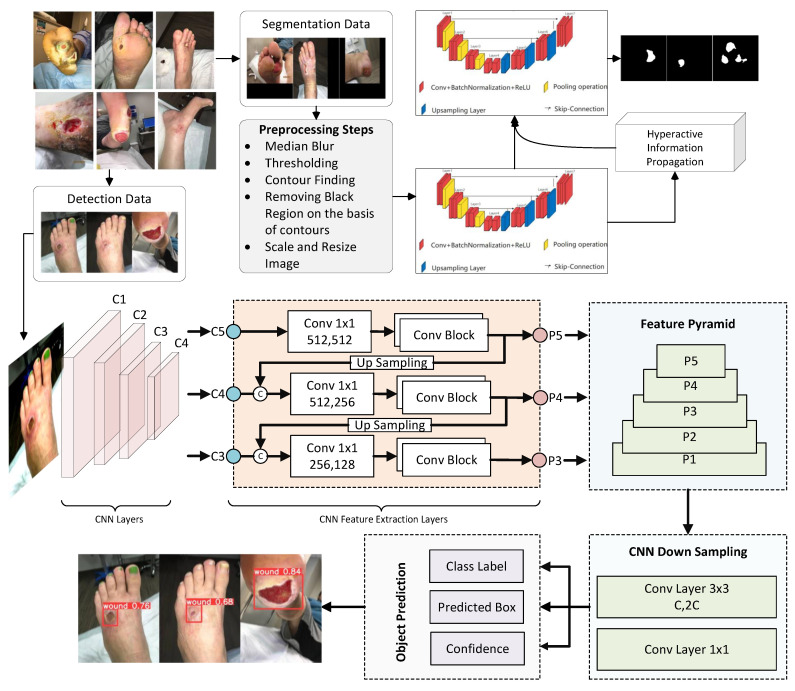
Working diagram of proposed CADFU system.

The process begins with an image with dimensions 512 × 512 pixels, containing troublesome black regions for training. Our initial task involves preprocessing to eliminate these black areas, which could be situated on the left, right, top, or bottom. Identifying the locations of these regions is the first step, followed by their removal through thresholding techniques. Subsequently, the primary image is extracted, resized, and normalized. These processes are briefly detailed in the ensuing sections. Following the initial preprocessing stages, we implement the DHuNeT algorithm. For detection, YOLOv8 is employed, taking advantage of the preprocessed data. In the case of detection, data preprocessing is absent, as the YOLO architecture itself incorporates basic preprocessing steps before applying the actual CNN layers. When an image is input, these two algorithms work in tandem, producing respective outcomes. YOLOv8 identifies wounds by enclosing them in red bounding boxes, while DHuNeT generates a segmentation mask for wound-specific regions. The operational framework of the proposed CADFU system is illustrated in Figure 1, while the subsequent paragraphs provide concise overviews of the algorithms.

### 3.1. Dataset Description

In this study, we employed the well-known FUSeg Challenge dataset for the segmentation and detection of foot ulcers. Specifically, we made use of two distinct datasets: the segmentation dataset and the AZH Wound Database for wound detection.

#### 3.1.1. Segmentation Data

**Figure 2:**
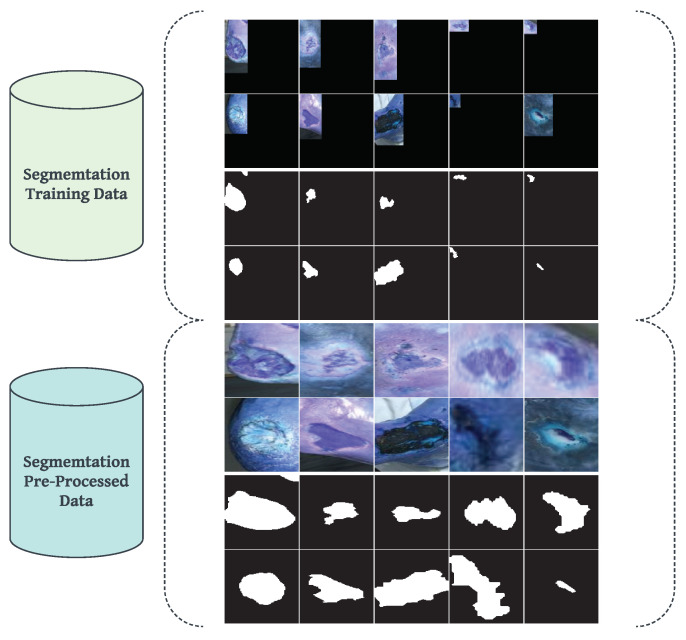
Unpreprocessed and preprocessed samples from segmentation data.

One pivotal aspect of the diagnosis and care protocol involves segmenting wound boundaries within images. This process holds significance as it aids in estimating wound area and providing quantitative measurements for effective treatment [7]. The image in Figure 2 displays samples from the segmentation dataset showcasing wounds.

The FUSeg Challenge persists as an ongoing benchmark for wound segmentation, offering a valuable resource for the medical community even after the conference’s conclusion [7].

#### 3.1.2. Detection Data

**Figure 3:**
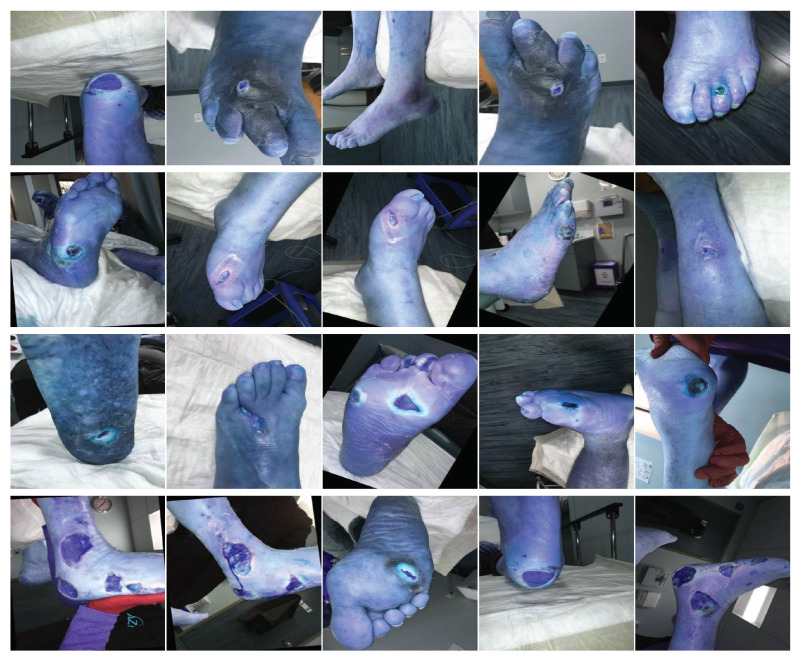
Unpreprocessed samples from detection data.

The AZH Wound Database, sourced from the AZH Wound and Vascular Center in Milwaukee, WI, USA, encompasses a collection of 1010 wound images [7,14]. The dataset encompasses three categories of ulcers: diabetic foot ulcer (DFU), pressure ulcer (PU), and venous ulcer (VU). The images have been captured using iPad and DSLR cameras without specific constraints on environmental or lighting conditions. Following capture, these images undergo further processing and are subsequently utilized for training and testing purposes [14]. The illustration in Figure 3 displays a representative sample from the dataset concerning wound detection.

To augment the dataset, ref. [14] applied various techniques to the AZH Wound Database. These techniques included rotations, flips (both vertically and horizontally), and blurring. As a result, the dataset expanded to a total of 4050 images. Within this dataset, 3645 images were designated for training, while the remaining 405 images were reserved for testing. Each image was subject to manual labeling to facilitate the training process and subsequent model evaluation. Table 1 shows the number of samples utilized for training and testing in wound segmentation and detection scenarios.

We use the 90:10 split because the dataset is limited and insufficient for training a large detection model like YOLO. Therefore, we allocate a larger portion of the dataset to the training phase, reserving only 10% (405 images) for testing. The results are very accurate and efficient. On the other hand, to enhance the model’s robustness, ref. [14] uses different data augmentation techniques to increase sample size. This reduces the risk of overfitting and contributes to the model’s ability.

### 3.2. Preprocessing Phase

In the preprocessing stage, we have developed a function that begins by transforming the provided image into grayscale using a median blur filter. This step improves the image’s readiness for further analysis. Various options for blur filters exist, with the Gaussian filter for blurring being represented by Equation (1):

(1) $$G_{0}\left(x,y\right)=A\cdot e^{-\frac{{\left(x-\mu_{x}\right)}^{2}}{2\sigma_{x}^{2}}-\frac{{\left(y-\mu_{y}\right)}^{2}}{2\sigma_{y}^{2}}}.$$

G(x,y) is the Gaussian distribution at point, σ represents the standard deviation of the Gaussian distribution, *e* is the base of the natural logarithm, and *x* and *y* are the coordinates in the image. Thresholding is then applied to create a binary image by setting pixel values to 255 or 0 based on intensity thresholds. The function proceeds with contour detection using the cv2.findContours() function, which identifies regions of interest in the binary image.

**Figure 4:**
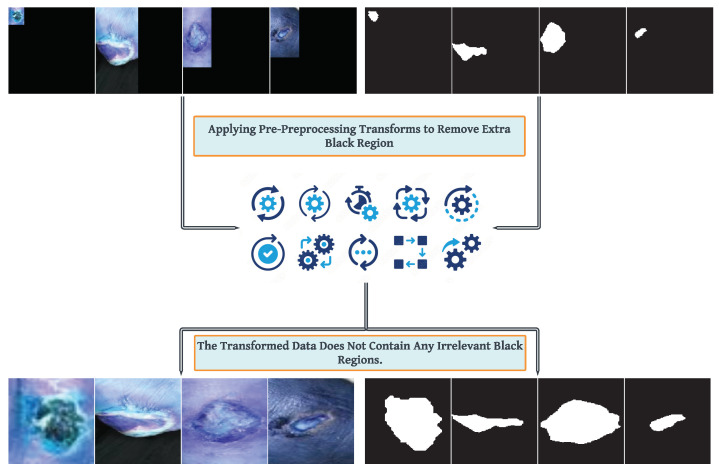
Preprocessing Results (Figure shows the Segmentation Data Before and After Preprocessing).

This preprocessing step aims to isolate the area containing the primary region of interest, presumably a wound in a medical context. The code iterates through the detected contours, calculating their areas and storing the contour with the largest area as the most prominent interest feature. This contour is subsequently approximated using the cv2.approxPolyDP() function to create a simplified version for further analysis. The approximated contour identifies a specific point farthest from the contour’s center using the far variable. Additionally, the coordinates of the maximum *x* and *y* values along the contour are captured, facilitating the calculation of the desired cropping dimensions. The final cropping is executed using these calculated dimensions, generating a new image and mask focusing solely on the region of interest. The visual representation depicted in Figure 4 illustrates the effects of various transforms on the original data, highlighting their role in enhancing the data into a more informative format. The Algorithm 1 shows the step-wise working of the proposed preprocessing strategy for data segmentation.
**Algorithm 1:** The Preprocessing Algorithm/Steps for Segmentation Task
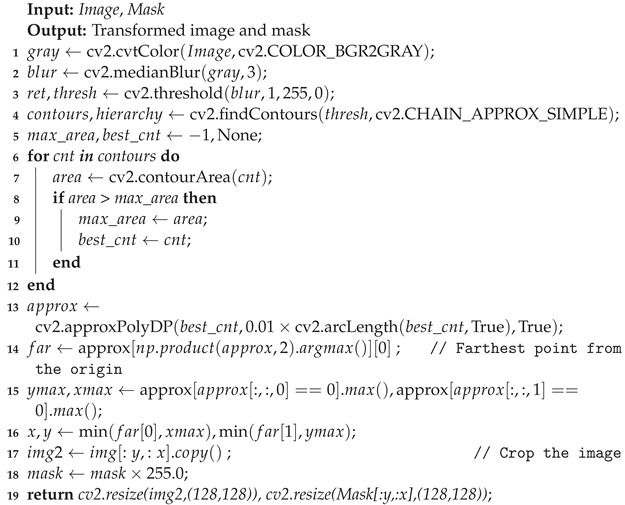


In the end, we resized both the cropped image and the mask to a consistent size of 128 × 128 pixels. This standardized size is often crucial for feeding data into neural networks, ensuring uniformity in training and analysis. The output of this function is a cropped and resized image, along with a correspondingly cropped and resized mask, both prepared for further stages of analysis or model training.

#### Functioning of DHuNeT (Dual-Phase Hyperactive UNet)

**Figure 5:**
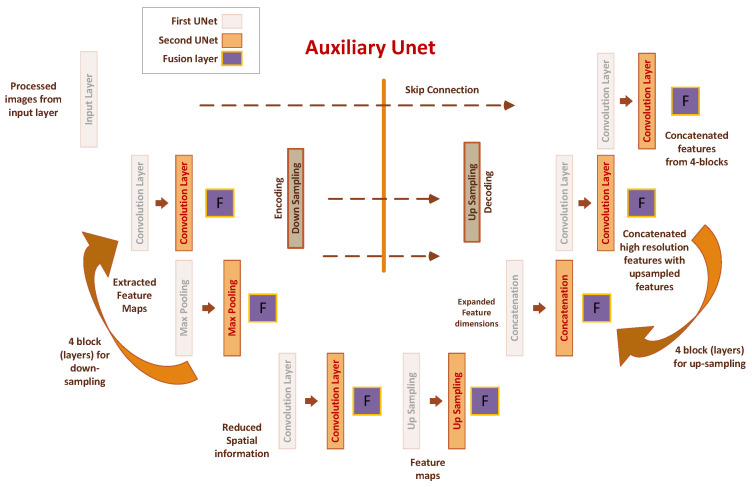
Proposed methodology of DHuNeT algorithm for wound segmentation.

As previously mentioned, we have designed a multitasking solution to address wound diagnosis, encompassing two distinct tasks. DHuNeT is one of the algorithms we have employed for segmentation. The operational dynamics of the proposed DHuNeT can be observed in Figure 5. In the subsequent sections, we delve into the functioning of DHuNeT, which integrates the UNet model. To begin, let us explore the UNet autoencoder.

The UNet autoencoder stands out as a pivotal architecture within the realm of image processing, renowned for its proficiency in medical image segmentation. Grounded in the principles of autoencoders, the UNet framework introduces an exceptional symmetrical and expansive design that allows it to uphold intricate details while learning meaningful features. Assuming X represents the input image, with ϕ as the encoder function and ψ as the decoder function, the mathematical representation of the basic autoencoder process is outlined in Equations (2)–(4).

(2) ϕ:X→F

(3) ψ:F→X

(4) $$\phi ,\psi =\underset{\phi ,\psi }{argmax}{∥X-\left(\phi \circ \psi \right)X∥}^{2}$$

At its heart, the UNet autoencoder is composed of two key elements: the encoder and the decoder. The encoder involves several convolutional and pooling layers that gradually reduce the spatial dimensions of the input image. This mechanism aids in capturing higher-level features through the gradual abstraction of representations. Conversely, the decoder employs techniques like upsampling and transposed convolutions to restore the original image dimensions while preserving contextual information. A hallmark of the UNet architecture lies in its incorporation of skip connections. These connections directly link corresponding layers between the encoder and decoder segments. By facilitating the transfer of low-level features from the encoder to the decoder, these connections enhance the model’s precision in localizing and segmenting objects in images. This attribute proves especially advantageous in medical imaging, where precise identification of structures holds paramount importance [15].

The UNet autoencoder has gained substantial traction in numerous medical image analysis contexts, notably in organ and tumor segmentation, as well as anomaly detection [16]. Its ability to capture intricacies while maintaining contextual information through skip connections positions it as a robust solution for scenarios requiring precise region delineation. The UNet autoencoder provides a robust framework for image analysis tasks, exemplifying its competence in medical image segmentation through its encoder–decoder configuration, the integration of skip connections, and its capacity to capture fine details while discerning high-level features.

This architecture consists of two main components: the downsampling path (encoder) and the upsampling path (decoder). The downsampling path is constructed using the downsample-block function, which applies two convolutional layers and pooling to progressively extract features. These features are then saved for later use in corresponding upsampling blocks. These extracted features are thoughtfully preserved to be utilized in the corresponding upsampling blocks at a later stage.

This architecture consists of two main components: the downsampling path (encoder) and the upsampling path (decoder). The downsampling path is constructed using the downsample block function, which applies two convolutional layers and pooling to extract features progressively. These features are then saved for later use in corresponding upsampling blocks. These extracted features are thoughtfully preserved to be utilized in the corresponding upsampling blocks at a later stage.

The second UNet is the same as the first one but also takes input information extracted by the first UNet model. The encoder of the second UNet takes features from the first UNet’s encoder, and the decoder of the second UNet takes features from the decoder of the first UNet, combining those features with their own extracted features. Let us assume each layer’s encoder and decoder features in the first UNet are denoted as $E_{i}^{1}$ and $D_{i}^{1}$, respectively. Similarly, for the second UNet, we have encoder and decoder features $E_{i}^{2}$ and $D_{i}^{2}$. These features are fused using the Equations (5) and (6). The $F_{i}^{e}$ and $F_{i}^{d}$ represent the fusion of features between the first and second UNet at each level *i*. All this fusion is performed at the second UNet level stage because, at that point, we have all the features from the first UNet, so we utilize them in the second UNet for improved performance efficiency.

(5) $$F_{i}^{e}=\left(E_{i}^{1}+E_{i}^{2}\right)/2$$

(6) $$F_{i}^{d}=\left(D_{i}^{1}+D_{i}^{2}\right)/2$$

A novel aspect is the fusion technique, which involves averaging the features of the primary UNet and an auxiliary UNet. This fused feature representation captures a collaborative understanding of the image’s features.

As the process progresses, the architecture iteratively fuses features from both UNets using the downsample blocks. This iterative fusion strengthens the collaborative context by combining the individual feature maps. Notably, this fusion approach enhances the network’s ability to capture multi-scale information.

After multiple fusion stages, a key point of interaction is introduced through the bottleneck layer. Here, features from both UNets (latent space features) are combined to capture shared and refined information. The use of an average function for feature fusion is consistent throughout the architecture. The final stages involve upsampling and feature reconstruction, wherein features are restored to their original dimensions. Fusion of these features, accomplished through average operations, ensures that the information from both UNets contributes to the final output. The architecture culminates in a Conv2D layer with sigmoid activation to produce the segmented output.

### 3.3. Functioning of YOLOv8

YOLOv8, which stands for “You Only Look Once Version 8”, is an advanced variant of the YOLO (You Only Look Once) object detection algorithm. YOLO algorithms are well-known for their real-time object detection capabilities in images and videos. YOLOv8 is designed to achieve even higher accuracy and speed by incorporating several architectural improvements [17]. At its core, YOLOv8 follows a single-pass approach, which means it processes the entire image just once to detect and locate objects within it. This design enables YOLOv8 to be remarkably efficient in terms of speed while maintaining accurate object detection. YOLOv8 employs a deep neural network architecture, typically based on convolutional neural networks (CNNs), to analyze the entire image simultaneously, generating bounding boxes around detected objects along with their associated class labels and confidence scores. The components in Equation (7) demonstrate the generalized loss function of the YOLOv8 model as introduced in the original YOLOv8 paper [17].

(7) $$L\left(\theta \right)=\frac{\lambda_{box}}{N_{pos}}L_{box}\left(\theta \right)+\frac{\lambda_{cls}}{N_{pos}}L_{cls}\left(\theta \right)+\frac{\lambda_{dfl}}{N_{pos}}L_{dfl}\left(\theta \right)+\phi {∥\theta ∥}_{2}^{2}$$

By utilizing Equation (7), ref. [17] designs the velocity term for the weight update rule as depicted in Equation (8).

(8) $$V^{t}=\beta V^{t-1}+\nabla_{\theta }L\left(\theta^{t-1}\right)$$

Through the utilization of Equation (8), the authors in [17] establish the primary rule for adjusting weights, incorporating the gradient term from Equation (8). The resulting equation is presented in (9).

(9) $$\theta^{t}=\theta^{t-1}-\eta V^{t}$$

The comprehensive loss function for YOLOv8 is outlined in Equation (10). These formulae are taken from the primary YOLOv8 research paper by Reis et al. [17].

(10) $$\begin{array}{c} L=\lambda_{\mathrm{box}}\frac{1}{N_{\mathrm{pos}}}\sum_{x,y}\sum_{c}\left(x,y\right.{{)}_{c}^{1}*\left(x,y\right.)}_{c}^{2}\rho^{2}+\alpha x,y\nu x,y^{3}\;\\ +\lambda_{\mathrm{cls}}\frac{1}{N_{\mathrm{pos}}}\sum_{x,y}\sum_{c\in \mathrm{classes}}y_{c}\log\left({\hat{y}}_{c}\right)+\left(1-y_{c}\right)\log\left(1-{\hat{y}}_{c}\right)\;\\ +\lambda_{\mathrm{df}\;l}\frac{1}{N_{\mathrm{pos}}}\sum_{x,y}\left(x,y\right.{)}_{c}^{1}h-\left(q(x,y)+1-qx,y\right.)\log\left({\hat{q}}_{x},y\right)\;\\ +\left(qx,y-q(x,y)-1\right.)\log\left(\hat{q}\left(x,y\right)+1\right)) \end{array}$$

where:

(11) $$qx,y=\mathrm{IoU}\left(x,y\right)=\frac{\beta \hat{x,y}\cap \beta x,y}{\beta \hat{x,y}\cup \beta x,y}$$

(12) $$\nu_{x,y}=\frac{4}{\pi^{2}}{\left(\arctan\left(\frac{w_{x,y}}{h_{x,y}}\right)-\arctan\left(\frac{{\hat{w}}_{x,y}}{{\hat{h}}_{x,y}}\right)\right)}^{2}$$

(13) $$\alpha_{x,y}=\frac{\nu }{1-q_{x,y}}$$

(14) $$y_{c}=\sigma \left(\cdot \right)$$

(15) $${\hat{q}}_{x,y}=\mathrm{softmax}\left(\cdot \right)$$

In the context of the YOLOv8 loss function (10), $N_{\mathrm{pos}}$ signifies the total count of cells that contain an object. The indicator function $1_{c}^{*}x,y$ denotes the presence of an object in specific cells. The tuple $\beta_{x,y}$ represents the ground truth bounding box with coordinates $\left(x_{\mathrm{coord}},y_{\mathrm{coord}}\right)$, width, and height. Similarly, $\beta \hat{x,y}$ corresponds to the predicted box for the respective cell. The tuple $b_{x,y}$ signifies the central point of the actual bounding box. The label $y_{c}$ denotes the true class label (not grid cell class) for each grid cell (x,y), regardless of object presence. The values q(x,y)+1 and q(x,y)−1 stand for the nearest predicted box IoUs on the left and right in $c^{*}x,y$. The parameters $w_{x,y}$ and $h_{x,y}$ refer to the boxes’ width and height. The variable ρ represents the diagonal length of the smallest enclosing box that encompasses both the predicted and ground truth boxes.

One of the distinguishing features of YOLOv8 is its utilization of skip connections, similar to those found in the UNet architecture. These skip connections allow the model to capture low-level and high-level features from various network layers. By combining these features, YOLOv8 detects both very small and closely placed objects with very high accuracy and precision. This is because the YOLOv8 model is trained on images of various sizes; therefore, it works well when multiple small and large objects are present in the image [17].

YOLOv8 is the best choice for various computer vision-related tasks, such as object recognition and multi-scale detection. YOLOv8 proves highly useful due to its fast processing speed, high accuracy, and multi-scale detection capability. Due to its remarkable speed, it is an effective solution for real-time and multi-scale object detection problems.

## 4. Results

In this section, we perform a comparative analysis with different state-of-the-art models presented in the literature. We also evaluate the proposed models by changing several factors; for example, in the detection case, we experiment with various image sizes, and in the segmentation case, we experiment with various loss functions. First of all, we analyze the key hyperparameters used in the proposed models for training, as shown in Table 2. After examining the hyperparameters, we briefly describe the dataset that is utilized in this research. The last subsections of this section show the result and visualization of obtained results and outcomes.

This analysis allows us to deeply scrutinize the outcomes of the proposed models with varying hyperparameters and adjustments, such as the number of filters or layers, essentially conducting an ablation study. Concluding this section, we present a comparative analysis involving state-of-the-art models documented in the existing literature. To code the overall architecture, we use the Python language because it provides many built-in libraries for deep learning. For training the wound diagnosis model, we use the Google Colab platform because it offers a free T4-GPU for fast computing. It is excellent for training larger vision models quickly and efficiently, providing 12 GB of RAM and 78 GB of disk space.

### 4.1. Comparative Analysis

Within this section, we conduct an in-depth comparative analysis by implementing different modifications to both data and models. In the context of wound segmentation, two separate training scenarios are explored: the first involves minimizing the binary cross-entropy loss function, and the second focuses on maximizing the Dice index (dice) by minimizing (1−dice). The outcomes of these variations are summarized in Table 3. Shifting our attention to detection tasks, we employ the YOLOv8 model and experiment with varying image sizes, including 128 × 128, 256 × 256, and 512 × 512 pixels. The findings of these experiments are detailed in Table 4. Subsequently, we visually present the outcomes and outputs generated by these models in both the segmentation and detection contexts. The crucial aspect is that all experiments were carried out using the same set of hyperparameter initializations. Two datasets are utilized in total, namely FUSeg segmentation and FUSeg detection. Therefore, the trained models are validated on the following testing samples: 278 testing samples for segmentation and 405 testing samples for detection.

#### 4.1.1. Comparative Analysis of Wound Segmentation

**Figure 6:**
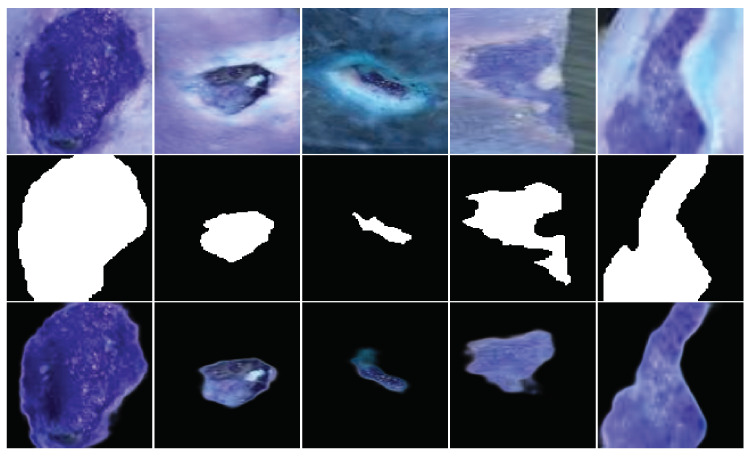

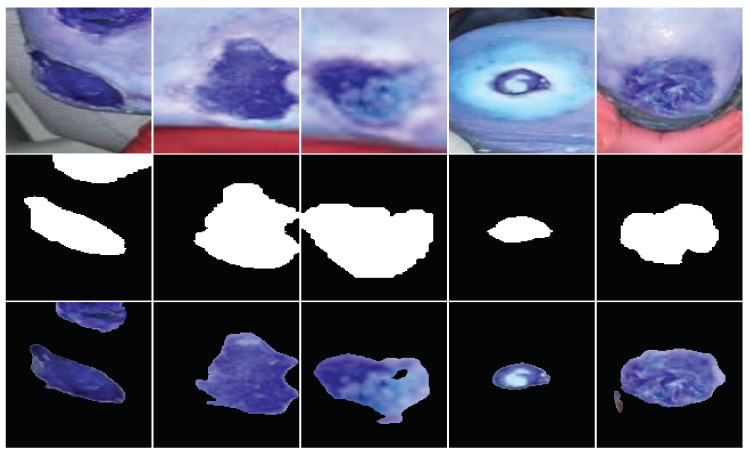
Results of segmentation model. (The upper subfigure displays the results using BCE loss, and the lower subfigure displays the results using Dice loss). In both figures, the top row displays the original images, the middle row showcases the predicted masks, and the bottom row exhibits the segmented regions.

As previously mentioned, our exploration of wound segmentation involves two distinct approaches. Initially, we employ binary cross-entropy detailed in Equation (16), as our loss function, and subsequently, we optimize the segmentation process by utilizing the Dice index function formulated in Equation (17). The outcomes of these segmentation strategies are visually presented in Figure 6 following the training process. Table 3 presents a comparison of outcomes between two segmentation scenarios.

(16) $$BCE\left(y,\hat{y}\right)=-\frac{1}{N}\sum_{i=1}^{N}y_{i}\log\left({\hat{y}}_{i}\right)+\left(1-y_{i}\right)\log\left(1-{\hat{y}}_{i}\right)$$

(17) $$Dice\left(y,\hat{y}\right)=\frac{2\sum_{i=1}^{N}y_{i}{\hat{y}}_{i}}{\sum_{i=1}^{N}y_{i}^{2}+\sum_{i=1}^{N}{\hat{y}}_{i}^{2}}$$

The metrics used for evaluating the DHuNeT model are Dice index, recall, precision, and F1-Score, described by Equations (17)–(20). The Dice index is very important because it describes the overlap between the actual and predicted wound mask. On the other hand, high recall means the model does not miss any important target regions in the segmentation case, while precision helps assess false positives. Lastly, the F1-Score shows the balance between recall and precision.

(18) $$\mathrm{Precision}=\frac{\mathrm{TP}}{\mathrm{TP}\;+\;\mathrm{FP}},$$

(19) $$\mathrm{Recall}=\frac{\mathrm{TP}}{\mathrm{TP}\;+\;\mathrm{FN}},$$

(20) $$F1-\mathrm{Score}=\frac{2\cdot P\cdot R}{P+R},$$

where R, P, TP, FP, and FN represent recall, precision, true positive, false positive, and false negative, respectively.

The analysis of the results in Table 3 highlights several key findings. In our case, the sample size of segmentation testing data is 278. When minimizing binary cross-entropy loss, the first UNet model (80.55% recall, 87.88% precision) outperforms the second (79.38% recall, 90.03% precision), indicating better precision for the latter. However, when maximizing Dice loss, the second UNet model (72.96% recall, 92.51% precision) performs slightly better than the first (75.03% recall, 92.60% precision). This suggests that the choice of loss function significantly impacts recall and precision, with the first UNet model showing stronger recall and the second excelling in precision. Overall, these findings underscore the trade-offs between different metrics when selecting segmentation strategies.

The outcomes of both models can be observed in Figure 6, representing the results from training two models with distinct loss functions. The top rows of Figure 6 display the original images, while the second rows illustrate the predicted masks. The segmented regions are displayed after the mask application in the final rows. These encouraging results underline the models’ effective feature learning capabilities.

**Figure 7:**
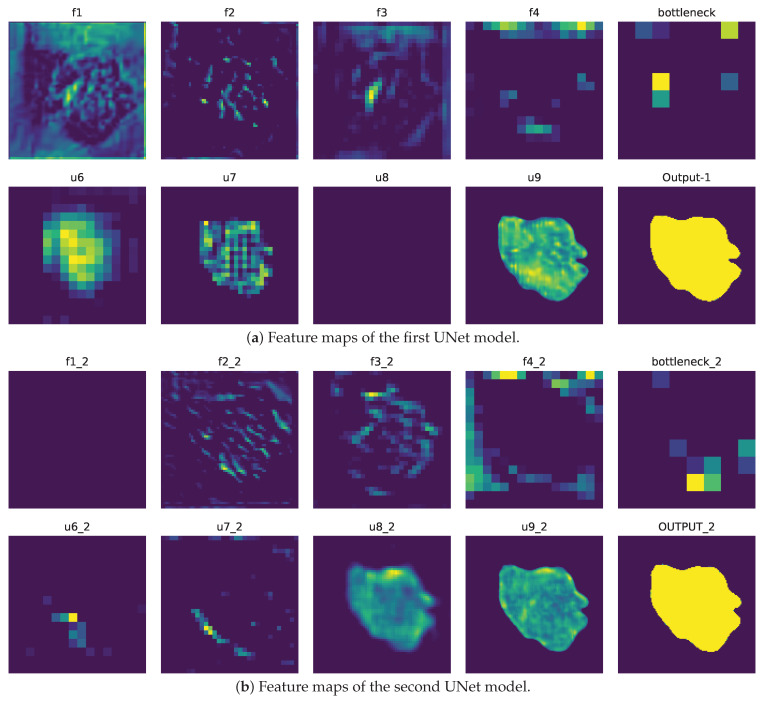
The illustration depicts the feature maps, showcasing how the model learns features and establishes the connection between images and masks.

Furthermore, the acquired features from the autoencoder are depicted in Figure 7, which is divided into two sections, symbolizing the collaborative nature of the stacked autoencoders called DHuNeT. Figure 7a showcases the features learned by the first UNet model, while Figure 7b portrays the features learned by the second UNet model. These visualizations effectively demonstrate the DHuNeT model’s strong ability to learn features effectively in both of its unique phases.

#### 4.1.2. Comparative Analysis of Wound Detection

Within this section, we concisely examine the YOLOv8 model’s performance, encompassing performance metrics and visual outcomes. Our initial step involves training the YOLOv8 model using multiple image sizes, ranging from 128 × 128 to 256 × 256 and 512 × 512 pixels. These results are meticulously outlined in Table 4. Subsequently, our analysis extends to the visual representation of the model’s efficacy in wound detection, providing a visual account of YOLOv8’s proficiency in accurately identifying wounds within images.

**Figure 8:**
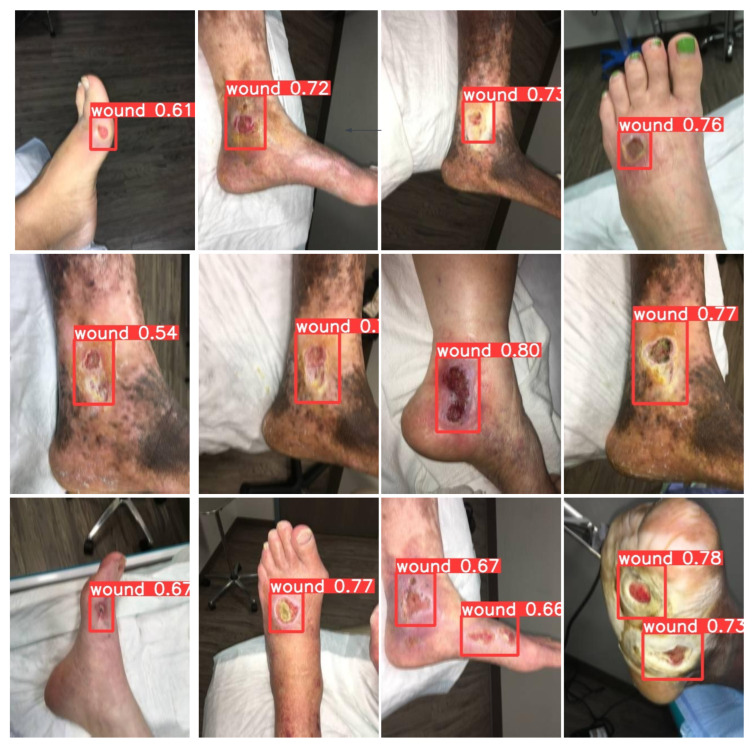
Results of the detection model.

The provided Table 4 offers an insightful comparison of wound detection performance metrics across different image sizes. Notably, an increase in image size from 128 × 128 to 512 × 512 pixels leads to improved recall and precision values. Additionally, the F1-Score exhibits a positive trend with increasing image size. This analysis indicates that larger image sizes contribute to enhanced detection performance in terms of recall, precision, and overall model accuracy. Multiple factors are responsible for why a larger image size contributes to the model’s performance. Firstly, higher image resolution is a key factor because larger images contain more pixels, meaning they have more in-depth detail. Secondly, smaller images may suffer from information loss during downsampling, whereas larger images reduce the risk of information loss. Thirdly, models trained on larger images are less prone to overfitting and are encouraged to generalize on the data. The outcomes of the model’s detection can be observed in Figure 8, illustrating a high level of precision.

### 4.2. Comparison with the State-of-the-Art

In this section, we conduct a comparison between the models we have introduced and the state-of-the-art models documented in existing literature. Our comparison is based on precision, recall, F1-Score, and Dice index metrics. We evaluate both the segmentation and detection models in comparison with the state-of-the-art alternatives, and the results are summarized in Table 5 and Table 6. The evaluation was performed against other contemporary models that were also trained on the same datasets.

**Figure 9:**
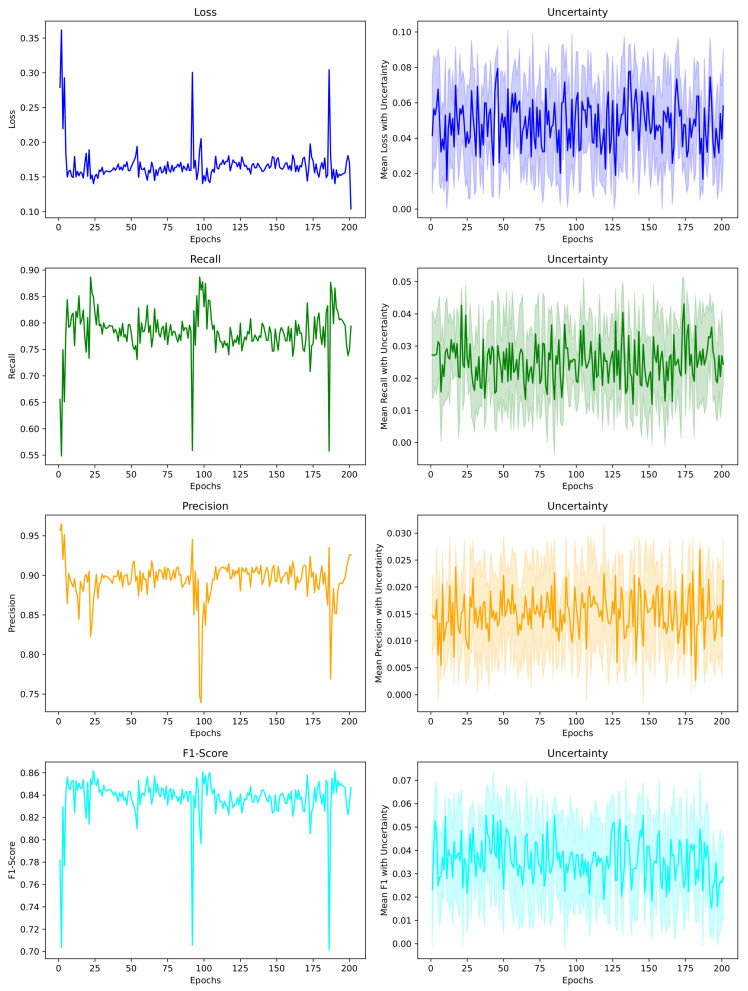
Performance metrics of the segmentation DHuNeT model (the x-axis represents the number of epochs, while the y-axis represents the performance metrics at each epoch). The first column shows the actual metrics, while the second column displays the metrics with uncertainty depicted in lighter colors.

The analysis presented in Table 5 comprehensively evaluates diverse segmentation models, assessing their performance based on various metrics. The VGG16 model of 2023 achieved a 78.35% recall and a Dice index of 81.03%, contrasting with the 2023 Ensemble model that displayed a notable precision of 92.23% and recall of 91.57%. The DFUSC model of 2023 showcased robust precision (84.56%) and an F1-Score of 76.96%. Conversely, the proposed DHuNeT model in 2023 showcased a well-rounded performance encompassing all metrics: 92.60% precision, 79.38% recall, 84.69% F1-Score, and an impressive Dice index of 85.0%. While some models excel in particular aspects, the DHuNeT model showcases a remarkable balance across all metrics. Its exceptional precision (92.60%) and high Dice index (85.0%) suggest that the model effectively captures the object boundaries without significantly over-predicting or under-predicting. This underscores DHuNeT’s overall performance superiority over other models despite their strengths in specific areas. Figure 9 shows the testing performance of the proposed DHuNeT segmentation model.

The Figure 9 displays the actual performance metrics along with their associated uncertainties in the second column. Uncertainty describes the degree of variability in the metric values. The values may fluctuate within the range indicated by the lighter colors in the second column of Figure 9.

The provided Table 6 presents a comprehensive comparative assessment of various detection models, highlighting their performance metrics. The Faster-RCNN model from 2015 demonstrated a substantial mean average precision (mAP) of 87.0%. In 2023, the ResNet50-FRCNN model exhibited competitive performance with a precision of 77.3%, recall of 89.0%, and an F1-Score of 82.7%, accompanied by a mAP of 71.3%. YOLOv5s, also in 2023, displayed a precision of 78.1%, recall of 68.5%, F1-Score of 73.2%, and a mAP of 76.9%. YOLOv4, introduced in 2022, showcased a mAP of 63.2%. The Faster-RCNN model of 2022 achieved a precision of 77.4%, recall of 64.0%, F1-Score of 69.0%, and a mAP of 70.9%. Notably, the proposed YOLOv8 model in 2023 yielded impressive precision (79.9%) and recall (74.5%), while also achieving a mAP of 86.0%. This comprehensive comparison underscores the advancements in detection models, with each model demonstrating varying strengths in achieving high accuracy, recall, and mAP scores.

**Figure 10:**
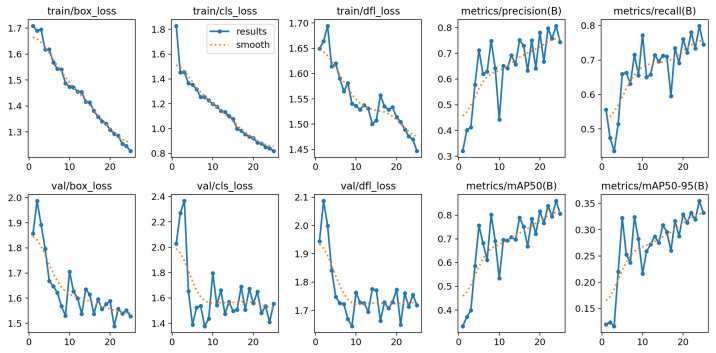
Performance metrics of YOLOv8 detection model (the x-axis shows the number of epochs, while the y-axis shows the metrics at each epoch).

The recall is crucial because, in segmentation tasks, it refers to efficiently and accurately identifying the relevant regions in images. Our proposed models have achieved very good and competitive recall scores. Our detection model exhibits high precision and mAP, showcasing its exceptional ability for accurate positive predictions. This is crucial for scenarios like medical diagnoses and security operations where minimizing false positives is vital. Figure 10 shows the overall performance metrics of the YOLOv8 detection model. The figure displays the training and validation losses and metrics.

Figure 10 displays the overall training and testing performance of the YOLOv8 model. Firstly, the box-losses and cls-losses in Figure 10 show the losses associated with the bounding box and class prediction; in our case, we have one class, which is wound. The dfl-loss evaluates object detection through the model’s localization and classification accuracy. The object detection precision and recall are shown by precision(B) and recall(B) plots. The term “mAP” refers to the mean average precision score. The mAP50 plot evaluates detection accuracy with an IoU threshold of 0.5, while mAP50-95 uses IoU thresholds from 0.5 to 0.95, providing a broader view of the model’s performance. We consider mAP50-95 as our performance metrics in Table 6. The “(B)” in parentheses in Figure 10 indicates the metrics are related to object detection on testing data. In our case, the testing and validation are the same.

The comparisons provided in Table 5 and Table 6 provide valuable insights into the performance of diverse segmentation and detection models. These tables emphasize the ongoing progress within their respective domains, as each model showcases specific strengths across various metrics. Significantly, the outcomes of the DHuNeT and YOLOv8 models stand out as promising, indicating exciting possibilities for future research and advancements in segmentation and detection methodologies.

## 5. Discussion

In this section, we discuss the real-world applications and some of the limitations of our proposed system. We also discuss the technological advancements in the healthcare sector. First of all, the key question is: is the proposed system useful for diagnosing other types of diseases? For example, is it useful for skin cancer or brain tumor diagnosis? The answer to this question is yes; it can be used for diagnosing other diseases after fine-tuning. If someone wants to use this system for skin cancer diagnosis, then it is compulsory to fine-tune our proposed system on a specific skin cancer dataset. Then the system can be used for skin cancer as well. It is important to note that the system is not useful for detecting multiple diseases at the same time.

The proposed work has many real-world applications in the healthcare sector, mainly in the medical imaging domain. The system can be used for real-time wound detection. The system is also integrated with the Internet of Things for automatic wound diagnosis. In the future, the system will also extend to wound assessments and monitoring, such as wound size and type identification. The proposed system can generally be used for efficient patient health monitoring.

Secondly, AI technologies have a significant impact on healthcare sectors in many different ways, such as diagnosing diseases, automating treatments, drug discovery, and the Internet of Medical Things (IoMT). More importantly, computer vision plays a very vital role in healthcare sectors. This is because, for diagnosing different diseases, doctors want to use different image modalities. For example, for identifying lung infections, they use X-rays, and for brain tumors, they use magnetic resonance imaging (MRI), and so on. Therefore, computer vision plays an important role in identifying the kind of chronic condition from image modalities.

In the last section, we mentioned some limitations of the proposed system. First of all, the system is trained on a specific dataset, so it may not perform as well in the real world. This is because every specific problem has specific constraints. Therefore, before applying the pre-trained models, the first task is to fine-tune and ensure that the system can be applied to that particular condition. Secondly, the models integrated into the systems have many parameters, so this may cause difficulties during deployment because it takes time in real-time inference.

Now, what are the solutions to these problems? In the future, researchers will try to reduce the parameters of models to make them more efficient and effective in real-world scenarios and less time-consuming. Secondly, researchers may train the system on a huge amount of data with diverse types of wounds for better generalization. In particular, if someone wants to use this system for a specific hospital, then it is advisable to fine-tune the system on the particular dataset of that hospital.

## 6. Conclusions

Efficient management of chronic wounds stands as a paramount concern in modern smart hospital environments. Among the commonly encountered wounds, foot ulcers hold a significant prevalence among patients. Timely intervention in wound treatment plays a crucial role in preventing its progression to a more severe condition. To address this critical concern, we present a pioneering Computer-Aided Diagnosis System for Foot Ulcers (CADFU) meticulously designed to offer both efficacious and proficient detection and segmentation of foot ulcers. Leveraging the prowess of YOLOv8 for ulcer detection and DHuNeT for ulcer segmentation, our research introduces the novel concept of DHuNeT, an abbreviation for Dual-Phase Hyperactive Knowledge UNet. This innovative system encompasses two core algorithms tailored for the precise execution of the detection and segmentation tasks. Our proposed system showcases remarkable efficacy, outperforming various state-of-the-art architectures. Operating as a valuable asset within smart hospitals, this system facilitates foot ulcer diagnosis and accelerates the treatment process.

## Figures and Tables

**Table 1:** Data samples for segmentation and detection.

Datasets	Training Samples	Testing Samples
Segmentation (FUSeg Data)	810	278
Detection (AZH Wound Data)	3645	405

**Table 2:** Hyperparameter values of models.

Model	Learning Rate	Batch Size	Trainable Parameters	Optimizer
**UNet**				
DHuNeT	0.005	32	1,081,942	Adam
**YOLOv8**				
YOLOv8	0.0001784	64	Default	Auto

**Table 3:** Wound segmentation (FUSeg dataset): performance metrics comparison for different scenarios.

Strategy	UNet	Recall	Precision	F1-Score	Dice Index
Minimize Binary Cross-entropy	1st-UNet	80.55	87.88	84.36	83.43
	2nd-UNet	79.38	90.03	84.69	83.96
Maximize Dice Index	1st-UNet	75.03	92.60	83.25	83.00
	2nd-UNet	72.96	92.51	81.96	82.00

**Table 4:** Wound detection: performance metrics comparison.

Image Size	Confidence	Recall	Precision	mAP
(128 × 128) pixels	0.25	74.3	71.2	81.0
(256 × 256) pixels	0.25	71.7	79.0	81.2
(512 × 512) pixels	0.25	79.9	80.8	86.0

**Table 5:** Comparison of segmentation models.

Model	Year	Test Samples	Metrics			
			**Precision**	**Recall**	**F1-Score**	**Dice Index**
VGG16 [18]	2023	200	83.91	78.35	81.05	81.03
Ensemble [8]	2022	200	92.23	91.57	91.9	84.09
SegNet [18]	2023	200	83.66	86.49	85.19	85.0
UNet [18]	2023	200	86.58	85.31	86.00	84.66
DFUSC [19]	2023	200	84.56	80.51	76.96	-
Feedforward NN [20]	2022	200	72.00	77.00	74.52	74.00
Proposed DHuNeT	2023	278	92.60	79.38	84.69	85.0

**Table 6:** Comparison of detection models.

Model	Year	Metrics			
		**Precision**	**Recall**	**F1-Score**	**mAP**
ResNet50-FRCNN [21]	2023	77.3	89.0	82.7	71.3
YOLOv5s [6]	2023	78.1	68.5	73.2	76.9
Faster-RCNN [22]	2022	77.4	64.0	69.0	70.9
Proposed YOLOv8	2023	80.08	79.90	79.95	86.0
